# Research progress on the relationship and mechanisms of obesity, gut microbiota, and natural medicines

**DOI:** 10.3389/fendo.2026.1804373

**Published:** 2026-08-07

**Authors:** Gaoman Chi, Yinghua Feng, Shanshan Jin, Jiaming Li, Jingyuan Ren, Rui Sun, Wenlin Zhang, Xiaoxuan Xu, Yangyang Liu

**Affiliations:** 1 School of Traditional Chinese Medicine, Changchun University of Chinese Medicine, Changchun, China; 2 Preventive Treatment Center, The Affiliated Hospital of Changchun University of Chinese Medicine, Changchun, China

**Keywords:** endocrine, gut microbiota, metabolic disorders, natural medicines, obesity

## Abstract

Obesity has become a major global public health challenge, affecting more than 1.5 billion people worldwide and markedly increasing the risk of chronic diseases, including type 2 diabetes mellitus (T2DM), cardiovascular diseases, and metabolic dysfunction-associated steatotic liver disease (MASLD). In recent years, the gut microbiota has emerged as a critical regulator of host metabolism, immune homeostasis, and energy balance and has been increasingly implicated in the development and pathogenesis of obesity. In obesity, gut microbial diversity often declines and community composition shifts (e.g., increased *Firmicutes/Bacteroidetes* ratio), which can disrupt energy homeostasis, promote lipid accumulation, trigger chronic inflammation, and alter appetite regulation. This review highlights evidence that natural medicines – including purified phytochemicals, standardized herbal extracts, and traditional multi-herb formulas – can mitigate obesity by beneficially reshaping the gut microbiota. Key mechanisms identified include restoration of microbial balance, increased production of short-chain fatty acids (SCFAs), enhancement of intestinal barrier integrity, and modulation of the gut–brain axis. However, most current evidence derives from preclinical studies; well-controlled human trials of microbiota-targeted natural therapies are still limited, and causal links between specific microbiota shifts and obesity outcomes remain to be established. Collectively, these findings provide a rationale for developing microbiota-oriented natural therapeutic strategies for obesity.

## 1 Introduction

Obesity has emerged as a major global public health challenge and represents a key risk factor for numerous metabolic disorders. Its prevalence continues to increase, and obesity-related metabolic complications are becoming increasingly severe. In this context, exploring the mechanisms underlying obesity and related metabolic diseases has become an area of intensive research. In recent years, considerable attention has been focused on the role of the gut microbiota in obesity pathogenesis and on therapeutic strategies targeting the intestinal microbiome. The human body is a complex ecosystem, and the gut microbiota, as its most important and active microecological component, is deeply involved in host metabolic and immune processes (1). Accumulating evidence indicates that gut microbiota dysbiosis and metabolic disturbances are closely interconnected, forming a vicious cycle that represents an important driving force in the development of obesity and metabolic abnormalities (2).

Meanwhile, natural medicines have demonstrated considerable potential in ameliorating obesity and associated metabolic dysfunction (3). In addition, studies have shown that natural medicines can reshape gut microbial composition and restore microbial homeostasis (4). In this review, the term “natural medicines” refers to naturally derived therapeutic agents, including purified natural compounds, standardized herbal extracts, single medicinal herbs, and traditional Chinese medicine (TCM) formulas. Because these categories differ substantially in their chemical composition, quality control, pharmacokinetic characteristics, and levels of clinical evidence, they are discussed separately throughout this review. Therefore, this review aims to elucidate the interplay between gut microbiota and obesity, summarize the feasibility and recent advances of natural products in improving obesity, highlight recent advances in the application of natural medicines for obesity management, and discuss future research directions and potential applications in this field.

## 2 Methods

A literature search was performed using PubMed and Web of Science to identify studies addressing the interactions among gut microbiota, obesity, and natural medicines. Studies published up to March 2026 were included, with particular emphasis placed on those published within the past five years. Highly cited landmark studies were also incorporated, where appropriate, to provide a historical perspective and contextualize key concepts.

The search strategy incorporated the following keywords and Medical Subject Headings (MeSH) terms: “obesity”, “gut microbiota”, “gut microbiome”, “intestinal microbiota”, “natural products”, “natural medicines”, “traditional Chinese medicine”, “short-chain fatty acids”, “gut–brain axis”, “intestinal barrier”, and “weight loss”. These terms were combined using the Boolean operators “AND” and “OR” to refine the search strategy.

Original studies, clinical trials, animal experiments, and review articles investigating the relationship between gut microbiota and obesity, as well as the role of natural products in obesity management through modulation of the gut microbiota, were eligible for inclusion. Publications unrelated to obesity, studies lacking microbiota-related outcomes, duplicate reports, conference abstracts, and non-English articles were excluded.

## 3 Relationship between gut microbiota and obesity

Obesity is a metabolic disease characterized by chronic excessive accumulation of adipose tissue, often accompanied by disorders of lipid, glucose, and protein metabolism, as well as chronic low-grade inflammation. According to recent estimates from the World Health Organization, more than 890 million adults worldwide were living with obesity in 2022, corresponding to approximately 16% of the global adult population (5), and its prevalence continues to rise annually. It not only seriously impairs physical and mental health and quality of life, but also imposes a substantial burden on healthcare systems. Therefore, weight reduction is a prerequisite for reducing the incidence of metabolic diseases and improving overall health. Evidence suggests that a 10% reduction in body weight can significantly improve various obesity-related chronic diseases (6).

The gut microbiota, as the “second genome” of the human body, comprises approximately 10 trillion microorganisms colonizing the intestinal tract. The number of microbial genes exceeds that of human genes by more than 150-fold (7). The human gut microbiota consists of several types of microbes including bacteria, archaea, eukarya, viruses, and microeukaryotes (8). At the phylum level, the gut microbiota is composed of *Bacteroidetes*, *Firmicutes*, *Actinobacteria, Proteobacteria*, and *Verrucomicrobia*, among which *Bacteroidetes* and *Firmicutes* are the most abundant (9, 10) and play key roles in host immunity and energy metabolism. The composition, function, abundance, and diversity of the gut microbiota influence human health (11) and are affected by multiple factors, including anatomical distribution, diet and lifestyle, medications, infections and inflammation, as well as genetic predisposition (12).

### 3.1 Key evidence that gut microbiota is an important regulator of obesity

The first direct evidence implicating the gut microbiota in obesity came from germ-free mouse studies. In 1983, Wostmann et al. found that germ-free mice required approximately 30% more caloric intake to maintain body weight compared with conventional animals (13), yet had lower body fat content. In conventional mice, total body fat was 42% higher than in germ-free mice, and gonadal fat was increased by 47%. Subsequent studies further confirmed that the transplantation of gut microbiota from conventional mice into germ-free mice led to a marked increase in body fat in the recipients, with a 60% increase occurring within a short period (14). This finding provided pioneering evidence that the gut microbiota serves as a factor influencing fat storage and obesity.

#### 3.1.1 *Firmicutes/bacteroidetes* ratio as a biomarker of obesity: a misconception and its reassessment

Early studies suggested that an increased *Firmicutes/Bacteroidetes* (F/B) ratio might represent a characteristic microbial signature of obesity. Ley et al. reported that the abundance of *Bacteroidetes* in obese animals decreased by about 50%, while the abundance of *Firmicute*s increased proportionally (15). Similar findings were subsequently observed in humans, leading to the hypothesis that the F/B ratio could serve as a potential biomarker for obesity. However, with the development of large-scale population studies and high-throughput sequencing technologies, the concept of the F/B ratio as a stable biomarker for obesity has increasingly been challenged. For example, a large population-based analysis using data from the American Gut Project showed that the F/B ratio in individuals with obesity was actually lower than that in the control group (16).In addition, systematic reviews and meta-analyses have demonstrated substantial heterogeneity across studies, indicating that no consistent or robust association exists between the F/B ratio and obesity (17).

First, gut microbiota composition is influenced by numerous factors, including dietary habits, age, sex, genetic background, geographical location, medication use, and lifestyle, resulting in substantial interindividual variability. Consequently, the F/B ratio may correspond to markedly different metabolic phenotypes (18). Moreover, obesity appears to be more closely associated with functional dysbiosis than with changes in microbial composition alone. Since distinct microbial taxa can perform overlapping metabolic functions, alterations in host metabolic phenotypes may occur even in the absence of significant changes in the F/B ratio. Furthermore, variations in sample collection procedures, DNA extraction methods, sequencing platforms, and bioinformatics pipelines may further compromise the reproducibility and comparability of findings (18). Therefore, compared with the F/B ratio, composite biomarkers based on functional microbial characteristics, microbial metabolites, and gut microbial ecological networks may possess greater predictive value.

### 3.2 Multidimensional associations between gut microbiota and obesity

Obesity has been associated with alterations in several specific microbial taxa, including *Christensenella, methanogens, Lactobacillus, Bifidobacterium*, and *Akkermansia* (19). The relative abundances of *Christensenella* and methanogens are negatively associated with body weight (20), whereas *Staphylococcus* and *Clostridium* species have been positively correlated with obesity. In addition, both *Akkermansia muciniphila and Akkermansia glycaniphila* have been shown to improve metabolic parameters in individuals with obesity. *Lactobacillus* and *Bifidobacterium* are essential for maintaining intestinal microbial homeostasis (21). Accumulating evidence suggests that individuals with obesity have reduced gut microbiota diversity (17). A low-diversity gut microbiota is closely associated with more severe obesity, higher levels of systemic inflammation, and more pronounced insulin resistance (22). Consistent with these observations, clinical studies have demonstrated that dietary and exercise interventions can increase microbial diversity in individuals with obesity, accompanied by improvements in metabolic parameters (23–25).

Differences in gut microbiota composition have also been observed among different obesity subtypes. Individuals with metabolically healthy obesity (MHO) show significantly higher levels of *Bacteroidetes* and *Lactobacillus*, while no significant difference in the F/B ratio is observed (26). In contrast, metabolically unhealthy obese (MUO) is characterized by increased abundances of *Prevotell*a and *Desulfovibrio* (27), together with reduced abundances of *Fusobacterium* and *Megamonas* (28). Notably, both MUO and MHO individuals exhibit higher levels of *Lactiplantibacillus plantarum* than metabolically healthy non-obese (MHNO) individuals (27).In addition, the gut microbiome has been associated with fat storage in different adipose tissue depots, suggesting that gut microbiota may be involved in regulating the distribution and function of distinct fat depots (29). Tavella et al.reported that higher abundances of *Christensenella, Rhodocyclaceae*, and Gram-negative bacteria may help reduce visceral adipose tissue, whereas decreased *Roseburia* abundance was positively correlated with the expression of several gene pathways in subcutaneous adipose tissue (30).In addition, the relative abundance of *Firmicutes* has been positively associated with brown adipose tissue markers in subcutaneous fat. These findings suggest that decreased *Roseburia* abundance and increased *Firmicutes* abundance contribute to enhanced subcutaneous fat accumulation (31). Nevertheless, the causal relationships between specific microbial signatures and regional adipose tissue development remain poorly understood.

A healthy gut microbiota is characterized by a diverse and balanced microbial ecosystem that performs essential physiological functions for the host. Disruption of this ecological balance, manifested by reduced microbial diversity, depletion of beneficial bacteria, or expansion of potentially pathogenic microorganisms, results in a state known as gut dysbiosis (32). Growing evidence indicates that gut dysbiosis is a key contributor to the initiation and progression of obesity. The underlying mechanisms cannot be explained solely by changes in the F/B ratio. The relationship between obesity and the gut microbiota involves overall microbial diversity, the functions of specific bacterial taxa, and heterogeneity in host metabolic phenotypes. Therefore, future research should move beyond taxonomic composition and focus on the integrated interactions among microbial functions, microbial metabolites, and host physiology. Such efforts may facilitate the development of microbiome-based strategies for the precise prevention and treatment of obesity.

## 4 Mechanisms by which gut microbiota regulate the development and progression of obesity

Targeted modulation of the gut microbiota represents a promising strategy for obesity management. Increasing microbial diversity and enriching beneficial bacteria have been shown to exert favorable effects on obesity and associated metabolic abnormalities (33). They can also regulate energy metabolism through microbial metabolites, such as SCFAs (34). Moreover, enrichment of beneficial microorganisms contributes to the restoration of intestinal barrier integrity, thereby limiting lipopolysaccharide (LPS) translocation into the circulation, blocking metabolic endotoxemia, alleviating metabolic inflammation, and ultimately improving obesity (35). In addition, the gut microbiota and its metabolites influence host appetite and energy intake through bidirectional communication along the gut–brain axis (36). These mechanisms act in a coordinated manner to regulate energy absorption, expenditure, and storage, as well as inflammatory and hormonal balance, thereby systematically improving host metabolic homeostasis and providing a scientific basis for microbiota-targeted interventions in obesity.

### 4.1 Restoration of gut microbiota homeostasis is a key mechanism in the regulation of obesity

Gut microbiota dysbiosis, characterized by reduced microbial richness and diversity, has been strongly linked to obesity and metabolic disorders (37). This dysbiosis mainly includes the loss of beneficial bacteria, excessive growth of harmful bacteria, and reduced microbial diversity. An imbalance of specific gut microbiota not only alters the microbial ecosystem but also affects metabolic functions, such as reducing the production of beneficial metabolites and compromising intestinal barrier integrity (38). As a consequence, increased translocation of endotoxins into the circulation promotes chronic low-grade inflammation and insulin resistance, thereby facilitating adipose tissue accumulation and weight gain. Maintenance of a stable gut microbial ecosystem is essential for preserving host metabolic homeostasis. Accordingly, correction of gut dysbiosis and restoration of microbial diversity are increasingly recognized as important therapeutic targets for obesity intervention.

### 4.2 Gut microbiota-derived metabolites are important regulators of obesity

The gut microbiota metabolizes undigested dietary components and host-derived substrates through microbial enzymatic and fermentation processes, generating a broad spectrum of metabolites. Although these metabolites are primarily confined to the intestinal lumen, some enter the systemic circulation and reach peripheral tissues, where they participate in the regulation of host energy metabolism, immune responses, and inflammatory processes. Among these metabolites, SCFAs are the most extensively studied. SCFAs mainly comprise acetate, propionate, and butyrate, which are present in the intestine at an approximate ratio of 1:1:3 (34). Most SCFAs are absorbed in the colon, whereas only 5%–10% are excreted in feces (34, 39).SCFAs play important roles in regulating metabolism, modulating host immune function, and maintaining intestinal barrier integrity. However, excessive SCFAs production has been associated with adverse outcomes, including an increased risk of liver cancer and kidney disease (40).

#### 4.2.1 Regulation of energy intake and satiety

Energy intake is regulated by multiple factors, among which SCFAs have emerged as important metabolic modulators. Their effects on appetite and energy homeostasis are mediated through multiple pathways involving energy storage, energy expenditure, intestinal barrier function, oxidative stress, and immune regulation. In particular, SCFAs stimulate the secretion of glucagon-like peptide-1 (GLP-1) and peptide YY (PYY) by activating FFAR2/FFAR3 receptors expressed on intestinal enteroendocrine L cells, thereby enhancing satiety and contributing to appetite regulation and energy homeostasis (41). These hormones suppress appetite and delay gastric emptying, ultimately leading to reduced caloric intake. Clinical studies further support the physiological relevance of this pathway. Supplementation with the propionate precursor inulin propionate increased satiety and reduced appetite (34). Additionally, a 24-week intervention study demonstrated that daily inulin propionate supplementation prevented weight gain in individuals with obesity and significantly reduced total body fat and visceral fat mass (42).

#### 4.2.2 SCFAs-mediated regulation of host metabolism and inflammation

SCFAs may prevent chronic low-grade inflammation by upregulating anti-inflammatory cells, reducing metabolic endotoxemia, and decreasing the production of pro-inflammatory adipokines and chemokines, while increasing energy expenditure, lipid oxidation, and lipolysis (43). Beyond their metabolic and immunomodulatory functions, SCFAs also contribute to intestinal homeostasis by promoting mucus secretion, enhancing the expression of tight junction proteins, and reducing intestinal permeability (44–46). These effects help limit the translocation of lipopolysaccharide (LPS), a major endotoxin derived from Gram-negative bacteria that links gut microbiota dysbiosis with obesity-associated chronic inflammation (47). Since restoration of intestinal barrier integrity represents an independent mechanism involved in obesity, this topic is discussed separately in the following section.

### 4.3 Restoration of intestinal barrier integrity is an important mechanism in the regulation of obesity

**Figure 1:**
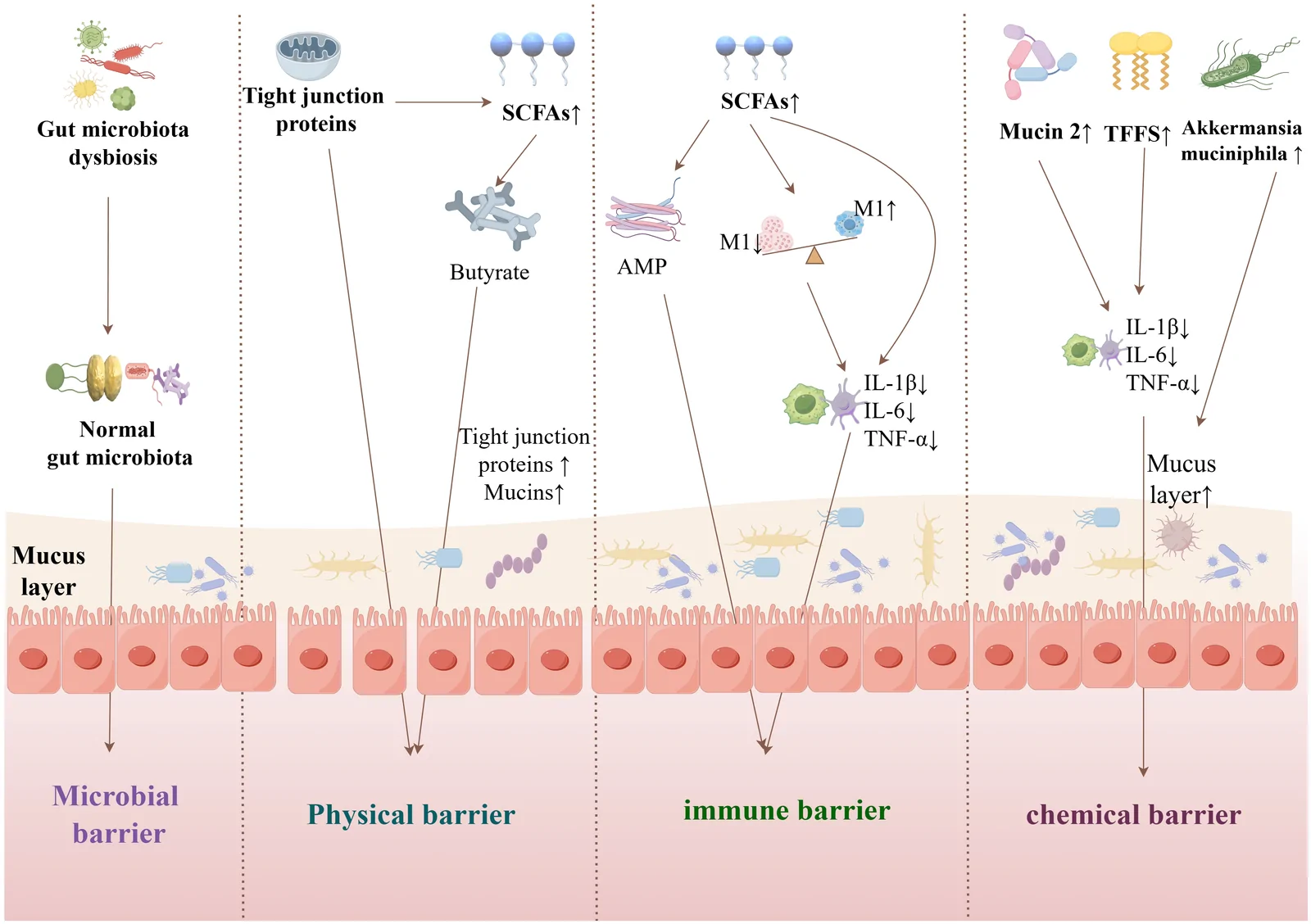
Interaction between the gut microbiota and the intestinal barrier. The figure depicts four aspects of the intestinal barrier: the microbial barrier, physical barrier, immune barrier, and chemical barrier. It explains the mechanisms of intestinal barrier impairment from these four perspectives.

The intestinal barrier is a multilayered defense system consisting of the physical, chemical, immune, and microbial components (48). Restoration of gut microbiota composition and ecological homeostasis contributes to the maintenance of the intestinal microbial barrier (48, 49) (**Figure 1**). The intestinal physical barrier primarily consists of intestinal epithelial cells interconnected by tight junction proteins. Disruption of epithelial integrity resulting from obesity, gut microbiota dysbiosis, or pathogenic insults increases intestinal permeability, allowing lipopolysaccharide (LPS) to translocate into the systemic circulation. Consequently, metabolic endotoxemia is induced, inflammatory signaling pathways are activated, and the progression of obesity-associated metabolic disorders is exacerbated (50) (**Figure 1**).

Mucin 2 (MUC2), the major structural glycoprotein of the intestinal mucus layer, represents a critical component of the chemical barrier. By preserving mucus integrity, limiting microbial invasion, and maintaining epithelial homeostasis, MUC2 is indispensable for intestinal barrier function and epithelial repair following injury (51). Consistently, mice deficient in MUC2 exhibit increased bacterial invasion of crypts and epithelial cells and display a higher susceptibility to inflammation and metabolic disorders (52).In addition, trefoil factor 3 (TFF3), another goblet cell-derived component of the mucus layer, contributes to mucosal repair. Increased expression of these protective factors enhances mucus layer thickness, reduces systemic pro-inflammatory cytokine levels, and facilitates restoration of the intestinal chemical barrier (**Figure 1**). The immune barrier comprises intestinal immune cells and antimicrobial molecules that coordinate host defense while maintaining immune tolerance. Gut microbiota-derived signals regulate antimicrobial peptide (AMP) production, immune cell differentiation, and macrophage polarization. In particular, suppression of pro-inflammatory M1 macrophages and promotion of anti-inflammatory M2 macrophages contribute to reduced production of IL-1β, IL-6, and TNF-α, thereby alleviating obesity-associated inflammation (53) (**Figure 1**).

### 4.4 The gut–brain axis is a key pathway in the regulation of obesity

**Figure 2:**
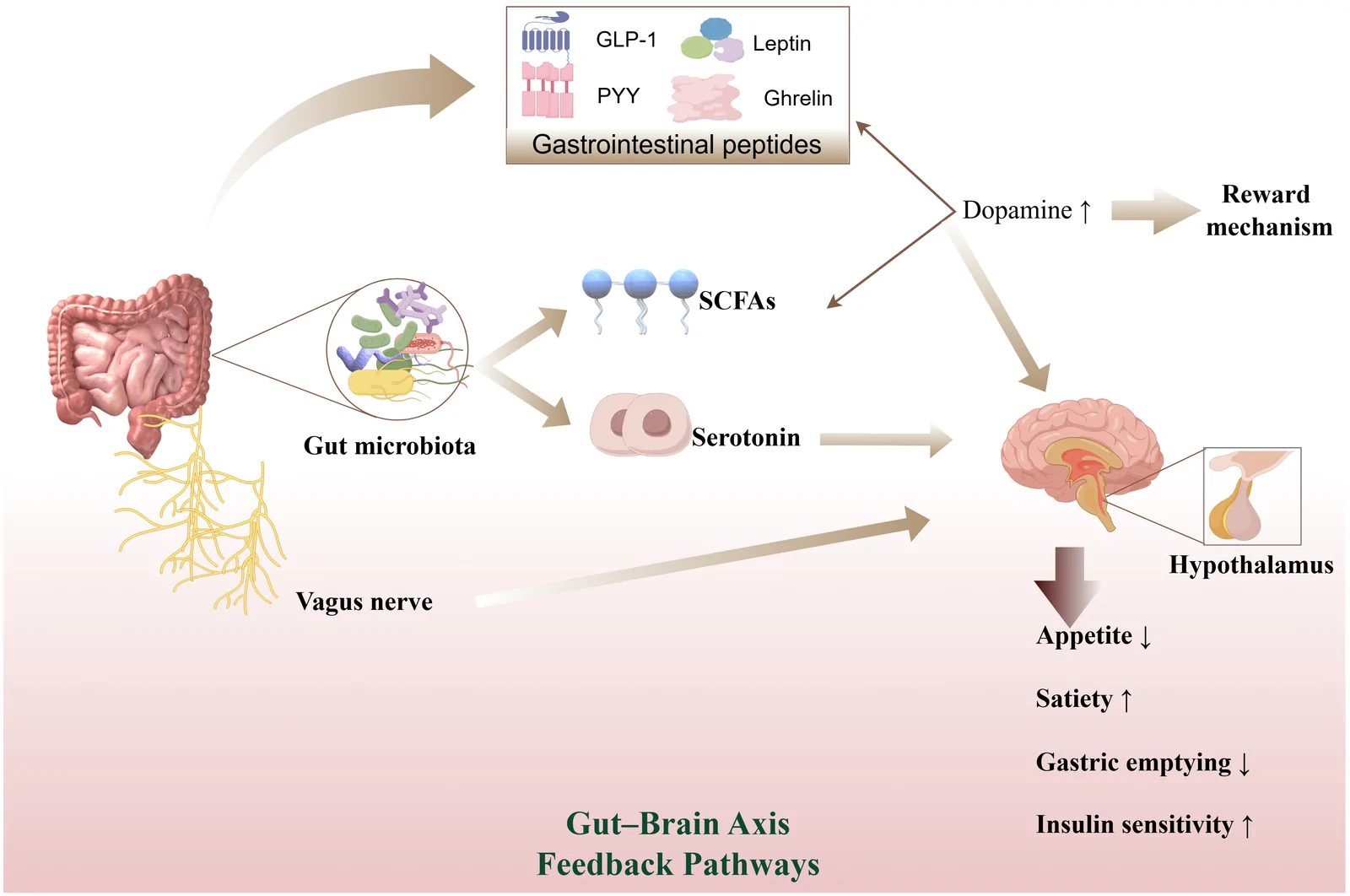
Gut–brain axis feedback pathway. The diagram illustrates the specific pathways of the gut-brain axis, including regulating the release of gut-brain peptides, the secretion of gut microbiota metabolites (SCFAs), and modulating appetite through reward mechanisms.

The gut microbiota and the brain communicate bidirectionally through a complex network collectively known as the “gut–brain axis.” This communication involves neural interactions, crosstalk with the immune and endocrine systems, and the synthesis and regulation of neuroactive substances (54–56). Within this network, the hypothalamus acts as a central regulator of food appetite and satiety. The vagus nerve serves as a major communication pathway linking the gastrointestinal tract and the central nervous system, transmitting information related to nutrient availability to the brain. Following food intake, nutrients stimulate enteroendocrine cells to release hormones, such as GLP-1 and PYY, which convey satiety signals to the central nervous system via vagal afferent fibers and contribute to the regulation of appetite and energy homeostasis (57) (**Figure 2**).

Gut microbiota-derived metabolites, particularly SCFAs, further participate in this regulatory network by stimulating the secretion of GLP-1 and PYY (58) (**Figure 2**). These hormones influence appetite and satiety by inhibiting neuropeptide Y (NPY), activating proopiomelanocortin (POMC) neurons in the arcuate nucleus (ARC) of the hypothalamus, or delaying gastric emptying (59, 60).In addition, NPY modulates insulin sensitivity and glucose homeostasis and regulates food intake, adiposity, and energy expenditure, thereby contributing to body weight regulation (61). The gut microbiota regulates gut–brain signaling through modulation of serotonin (5-hydroxytryptamine, 5-HT) synthesis and metabolism. Approximately 90% of circulating 5-HT is produced by enterochromaffin cells in the intestine, and its secretion is influenced by both gut microbiota and their metabolites (62) (**Figure 2**). Furthermore, the gut microbiota and its metabolites influence neurotransmitter systems and dopamine pathways, thereby modulating food reward responses (63) (**Figure 2**). The brain reward system, often referred to as the “pleasure–reinforcement” network, plays a pivotal role in feeding behavior. Consumption of highly palatable foods, gut microbiota dysbiosis, and specific microbial metabolites can alter dopamine release and activate reward pathways. These pathways interact with gut-related hormones such as ghrelin and leptin. During fasting, ghrelin enhances the responsiveness of reward pathways to food-related stimuli, thereby increasing appetite. In contrast, obesity-associated leptin resistance results in persistent activation of reward signaling, which may contribute to excessive food intake. Importantly, the relationship between reward dysfunction and obesity appears to be bidirectional. Altered reward processing promotes overeating and weight gain, whereas obesity itself further impairs dopaminergic signaling, reinforcing food-seeking behaviors and perpetuating a vicious cycle.

## 5 Research progress on natural medicines regulating gut microbiota in the control of obesity

**Figure 3:**
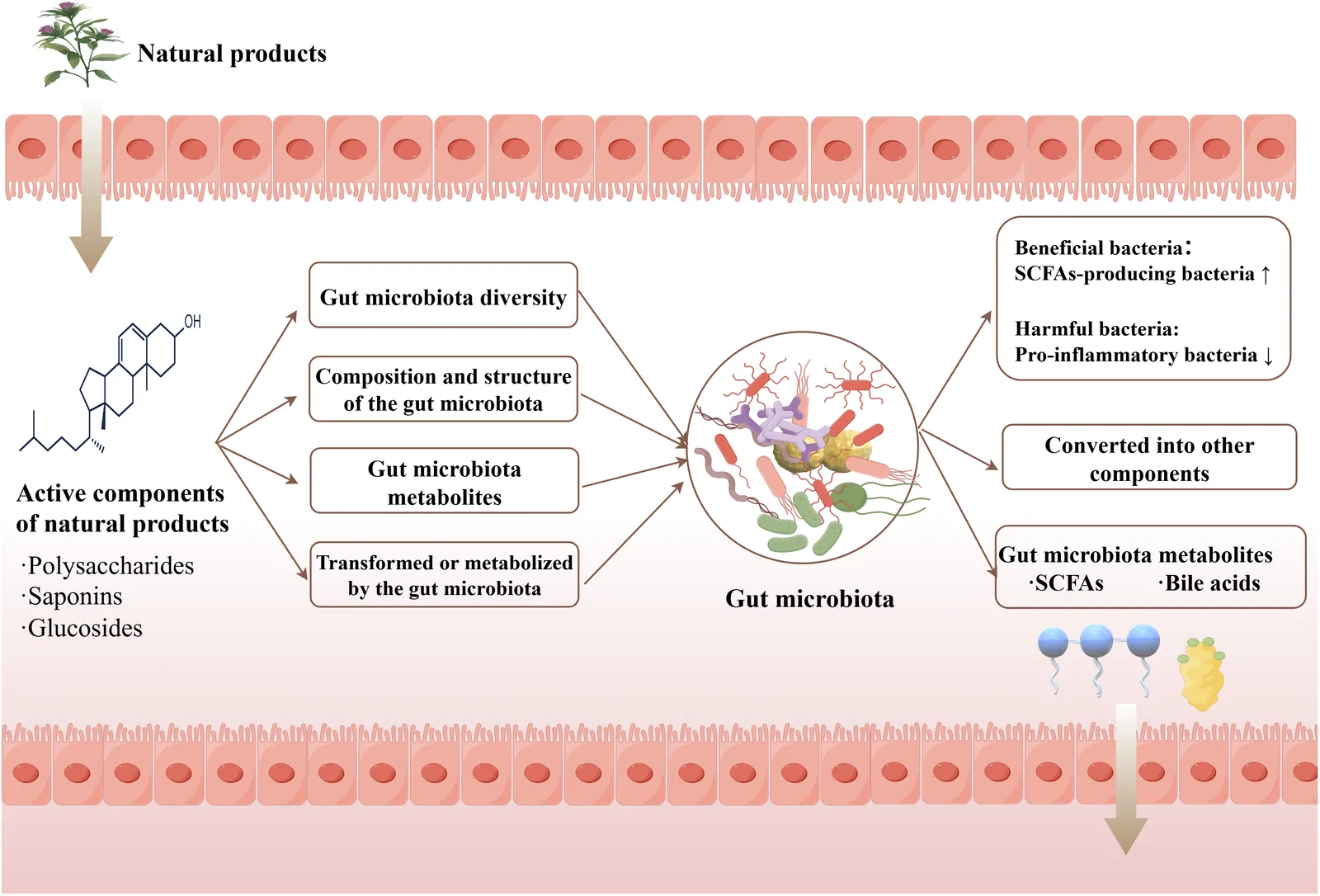
The relationship between natural medicines and the gut microbiota. The figure illustrates how active components in natural medicines restore gut microbiota diversity and regulate the gut-brain axis by modulating the structure of the gut microbiota and the secretion of gut microbiota metabolites. This improves gut microbiota function, thereby achieving weight loss and alleviating metabolic disorders.

In recent years, increasing attention has been focused on the interactions between gut microbiota and natural medicines. Natural medicines exert anti-obesity effects through multiple mechanisms, including appetite suppression, reduced energy intake, increased energy expenditure, regulation of lipid metabolism, and reversal of obesity-related gut microbiota dysbiosis (3). The gut microbiota can influence the intestinal transformation and absorption of some natural medicines, thereby improving their bioavailability and therapeutic efficacy. Moreover, some natural medicines exert their pharmacological effects through specific microbial communities (64). Gut microorganisms are also capable of converting natural compounds into bioactive metabolites, further enhancing their bioavailability (65) (**Figure 3**). For example, polysaccharides can be fermented by gut microbiota to produce SCFAs. In turn, natural medicines can directly reshape gut microbial composition and modulate the production of microbial metabolites (66) (**Figure 3**). For instance, administration of *Pueraria lobata* in mice with microbiota-related diarrhea resulted in a reduction in SCFAs levels (67).

Natural medicines have also been shown to enhance intestinal barrier function (**Figure 3**). Rhein anthraquinone glycoside preparation (RAGP) (68) improves intestinal barrier integrity by increasing the abundance of mucin-degrading bacteria and upregulating the expression of tight junction and adhesion proteins, thereby reducing inflammation and contributing to metabolic improvements associated with obesity. The gut–brain axis is considered an important pathway through which natural medicines exert their effects, as many natural medicines stimulate GLP-1 secretion to regulate gut–brain signaling (69) (**Figure 3**). Importantly, the effects of natural medicines on gut microbiota and obesity are not mediated by individual compounds alone but arise from the coordinated actions of multiple components acting on multiple targets (3). The drug combinations and compound formulas (prescriptions) often enhance efficacy, reduce toxicity, or generate new functions, producing therapeutic effects that exceed those of single herbs.

### 5.1 Bioactive compounds isolated from medicinal herbs

#### 5.1.1 Berberine

Berberine (BBR) is the major isoquinoline alkaloid isolated from the traditional medicinal herb *Coptis chinensis*. Although the whole herb contains multiple bioactive constituents, current evidence indicates that berberine is the principal compound responsible for its anti-obesity and gut microbiota-modulating activities. BBR is considered a key component responsible for gut microbiota regulation and metabolic improvement (70, 71). BBR has been shown to inhibit adipocyte proliferation and differentiation by downregulating the expression of obesity-related genes (72). Berberine can activate the AMPK signaling pathway, enhance glucose uptake and lipid metabolism–related protein expression, and thereby suppress adipose tissue expansion (73). Accumulating evidence indicates that BBR exerts beneficial metabolic effects through modulation of the gut microbiota. Specifically, BBR increases the abundance of beneficial microorganisms while suppressing potentially harmful bacteria (74, 75). For example, BBR can increase the abundance of beneficial bacteria such as *Akkermansia muciniphila*, thereby improving gut microbiota composition, enhancing intestinal barrier function, and alleviating obesity-related metabolic disorders (76). BBR has also been reported to regulate the gut–brain axis and HFD-induced metabolic disorders (53). In experimental studies, BBR increased gut microbial diversity and altered the relative abundances of *Bacteroidetes* and *Firmicutes*, accompanied by elevated serum levels of GLP-1, GLP-2, PYY, glucose-dependent insulinotropic polypeptide (GIP), and growth hormone-releasing peptide (77). These changes contribute to improvements in obesity and obesity-related metabolic disorders and support microbiota-mediated regulation of the gut–brain axis (78). Moreover, increased concentrations of SCFAs following BBR treatment have been linked to enhanced intestinal barrier function (79) (**Table 1**). BBR may also improve therapeutic efficacy by regulating drug bioavailability in individuals with obesity (64, 80).

**Table 1:** Summary of preclinical studies investigating the anti-obesity effects of natural medicines through modulation of the gut microbiota.

Category	Representative agent	Source	Experimental model	Mode of action	References
Purified natural compound	Berberine	*Coptis chinensis*	HFD-induced obese mice	*• Akkermansia muciniphila, Bacteroides, Bifidobacterium, Lactobacillus, Faecalibacterium, and Roseburia*↑ • *Desulfovibrio, Escherichia–Shigella, and F/B ratio*↓ • Regulates gut hormones: GLP-1, GLP-2, PYY, GIP, and ghrelin; *Regulates the gut-brain axis; • Improves intestinal barrier integrity	(105) (106)
Standardized herbal extract	Ginseng extract	*Panax ginseng*	HFD-induced obese mice	• *Akkermansia muciniphila, Bacteroides, Faecalibacterium, Ruminococcus, Bifidobacterium*↑ • F/B ratio↓ • Regulates the gut-brain axis; • Improves intestinal barrier integrity • Regulates bile acid metabolism, regulates SCFAs production	(88) (107)
Purified natural compound	Puerarin	*Pueraria lobata*	HFD-induced obese mice	• *Akkermansia muciniphila, Lactobacillus*, and SCFAs-producing bacteria↑ • F/B ratio ↓ • modulates SCFAs and bile acid metabolism • modulates the gut-brain axis	(97) (92)
TCM formula	Gegen Qinlian Decoction	Formula	HFD-induced obese mice	• *Bacteroidetes*, *Akkermansia muciniphila*↑ • *Lactobacillus*↓ • Modulates SCFAs and bile acid metabolism • Improves intestinal barrier integrity	(100)
TCM formula	Sijunzi Decoction	Formula	HFD-induced obese mice	• *Bifidobacterium, Lactobacillus*↑ • Improves intestinal barrier integrity • modulates SCFAs	(103)

Numerous animal studies have demonstrated that BBR alleviates obesity through modulation of the gut microbiota. In HFD-induced obese mice, a 14-week BBR intervention significantly increased the abundance of *Akkermansia muciniphila*, accompanied by reduced adipose tissue accumulation, attenuation of adipose inflammation, and improved insulin resistance (81). Similarly, 12 weeks of BBR treatment significantly increased SCFA levels, which were associated with enhanced intestinal barrier integrity and improved metabolic dysfunction (82). Clinical evidence also supports the beneficial metabolic effects of BBR. In a multicenter, randomized, double-blind, placebo-controlled trial involving 409 participants, 12 weeks of BBR treatment significantly reduced body weight and waist circumference in patients with diabetes. Metagenomic analyses further demonstrated that BBR reshaped gut microbial composition, increased the abundance of beneficial bacteria, and ameliorated gut microbiota dysbiosis (83). However, high-quality clinical evidence directly linking the anti-obesity effects of BBR to gut microbiota modulation remains limited. Therefore, the mechanisms observed in humans require further confirmation in large-scale randomized controlled trials.

Berberine possesses both hypoglycemic and weight-reducing properties. These effects are mediated through multiple mechanisms, including improvement of insulin resistance, remodeling of gut microbiota composition, attenuation of oxidative stress, and suppression of chronic low-grade inflammation (83). Furthermore, BBR promotes the enrichment of SCFAs-producing microorganisms, enhances intestinal barrier integrity, and reduces intestinal permeability, thereby lowering the risk of metabolic endotoxemia and contributing to glucose homeostasis (83) (**Table 2**). These findings suggest that BBR represents a promising therapeutic option for obesity accompanied by type 2 diabetes mellitus.

**Table 2:** Summary of clinical studies investigating the anti-obesity effects of natural medicines through modulation of the gut microbiota.

Category	Representative agent	Source	Participants	Mode of action	References
Purified natural compound	Berberine	*Coptis chinensis*	409 patients with diabetes	• *Blautia, Bacteroides, and γ-Proteobacteria*↑ • *bacteria associated with endotoxemia*↓ • modulates bile acid metabolism	(83)
Standardized herbal extract	Ginseng extract	*Panax ginseng*	10 obese middle-aged Korean women	• *Blautia, Faecalibacterium, and Anaerostipes*↑	(89)

#### 5.1.2 Ginsenosides and ginseng extract

*Panax ginseng* contains major bioactive constituents, including ginsenosides, polysaccharides, volatile oils, and glycosides (84). Ginseng extract (GE) can increase the abundance of *Enterococcus faecalis*, promoting the release of long-chain fatty acids, especially myristic acid (85), which in turn enhances brown adipocyte activity and beige fat formation, thereby alleviating obesity. GE also improves metabolic dysfunction by regulating gut microbial composition and preserving intestinal barrier integrity. Specifically, GE increases microbial diversity, reduces the F/B ratio, promotes the growth of beneficial bacteria such as *Parabacteroides, Akkermansia, and Ruminococcus*, and suppresses harmful bacteria such as *Helicobacter pylori* and *Lachnospiraceae members* (86). In addition, GE enhances the expression of tight junction proteins and tubulin, thereby maintaining intestinal epithelial integrity and promoting intestinal health (87).

Numerous animal studies have demonstrated that ginseng and its bioactive components exert significant anti-obesity effects and may improve host metabolic status by modulating the gut microbiota. In an HFD-induced obese mouse model, treatment with ginsenoside Rb1 alleviated obesity-associated metabolic disorders by reshaping gut microbiota composition, increasing the abundance of beneficial bacteria such as *Akkermansia muciniphila* and *Parasutterella*, regulating long-chain fatty acid metabolism, and improving insulin resistance and lipid abnormalities (88) (**Table 1**). Clinical evidence is also emerging. In a study conducted by Song et al., ten obese middle-aged Korean women received GE for 8 weeks. Compared with baseline values, treatment significantly reduced body weight and BMI and was accompanied by marked alterations in gut microbiota composition, mainly involving changes in the abundances of *Blautia, Faecalibacterium*, and *Anaerostipes* (89) (**Table 2**). Collectively, these findings suggest that ginseng exerts anti-obesity and metabolic regulatory effects through modulation of the gut microbial ecosystem. However, clinical evidence supporting the anti-obesity effects of ginseng remains limited, and most studies have focused on overweight individuals or individuals with obesity accompanied by impaired glucose metabolism.

Obesity is commonly accompanied by dyslipidemia, chronic low-grade inflammation, and cardiovascular metabolic abnormalities. Previous studies have shown that ginseng and its ginsenosides increase the abundance of probiotics and SCFAs-producing bacteria, improve intestinal barrier integrity, and attenuate inflammatory responses, thereby ameliorating lipid metabolic disorders and reducing the risk of atherosclerosis (90). Therefore, ginseng and its active constituents may provide therapeutic benefits for individuals with obesity and cardiovascular metabolic abnormalities through modulation of the gut microbiota, attenuation of inflammation, and improvement of lipid metabolism.

#### 5.1.3 Puerarin

Puerarin, the principal bioactive constituent of *Pueraria lobata*, belongs to the isoflavone family and exhibits a wide range of biological activities. It has been widely used in complementary and alternative medicine for the management of various metabolic disorders (91). Puerarin exerts anti-obesity effects through multiple mechanisms, including regulation of energy homeostasis, improvement of glucose and lipid metabolism, attenuation of insulin resistance, and modulation of the gut microbiota (92). It inhibits lipogenic enzyme activity in adipose tissue, reduces oxidative stress and chronic inflammation, suppresses adipocyte proliferation and differentiation, and improves obesity-related metabolic disorders. In terms of gut microecology, puerarin mainly regulates microbial composition, restores microbial diversity, and increases beneficial bacteria, thereby exerting anti-obesity effects. In obese mice, puerarin increased SCFAs levels in intestinal contents and enhanced intestinal barrier integrity, indicating improved microbial metabolic function (93). Furthermore, puerarin has been shown to reduce intestinal lipid absorption via the brain–gut axis by acting on jejunal microvilli and the dorsal motor nucleus of the vagus nerve (94). Persistent expansion of white adipose tissue induces endoplasmic reticulum stress and promotes the formation of small lipid droplets, eventually leading to cell necrosis, inflammation, extracellular matrix deposition, and fibrosis (95). Emerging evidence suggests that puerarin-induced alterations in the gut microbiota are associated with reduced white adipose tissue mass in mice (96).

Currently, evidence supporting the anti-obesity effects of *Pueraria lobata* and its bioactive constituents through modulation of the gut microbiota is derived predominantly from animal studies, and clinical data remain limited. Wang et al. employed an HFD-induced obese mouse model and found that puerarin intervention significantly attenuated body weight gain, improved insulin resistance and inflammatory status, increased the abundance of *Akkermansia muciniphila*, and upregulated the expression of intestinal barrier-related proteins, including Muc2, ZO-1, and Occludin (97) (**Table 1**). These effects collectively alleviated obesity-associated metabolic abnormalities through reshaping gut microbiota composition and restoring intestinal barrier function. Collectively, current evidence suggests that *Pueraria lobata* exerts anti-obesity effects through the gut microbiota–metabolism axis. However, its long-term efficacy and potential mechanisms in humans require further investigation.

Puerarin can reduce blood glucose levels by promoting insulin secretion and improving metabolic function. In addition, puerarin has been reported to promote pancreatic β-cell proliferation through upregulation of glucagon-like peptide-1 receptor (GLP-1R) and enhancement of GLP-1R signaling pathways (98). Given its potential effects on body weight, glucose metabolism, and insulin sensitivity, *Pueraria lobata* and its bioactive constituents may represent promising therapeutic option for individuals with obesity accompanied by type 2 diabetes mellitus.

### 5.2 Holistic regulation of traditional Chinese medicine

#### 5.2.1 Gegen Qinlian decoction

Gegen Qinlian Decoction(GQD) is mainly composed of *Pueraria lobata* and *Coptis chinensis*. Multiple studies have confirmed that this formula can significantly improve body weight, visceral fat index, glucose and lipid metabolism, and insulin resistance by alleviating inflammation and modulating gut microbiota. GQD and its major bioactive components increase the abundance of beneficial gut bacteria while reducing pathogenic microorganisms, promote SCFAs production, restore intestinal barrier integrity, and reduce LPS translocation into systemic circulation, thereby suppressing systemic inflammatory responses (99). In obese mouse models, GQD treatment increased the abundance of *Bacteroidetes*, *Akkermansia*, and *Muribaculaceae*, whereas the abundance of *Lactobacillus* was decreased (100) (**Table 1**). Zeng Guowei and colleagues further reported that GQD regulates SCFAs-producing bacteria and influences multiple metabolic pathways, while also restoring intestinal barrier function and reducing LPS translocation into the bloodstream (99). In addition, the major active components of GQD, including Berberine and Baicalin, may regulate bile acid metabolism through modulation of the gut–brain axis (99).

#### 5.2.2 Sijunzi decoction

Sijunzi Decoction is mainly composed of herbs such as *Panax ginseng* and Poria cocos and exerts immunomodulatory effects by regulating gut microbiota and SCFAs levels (101, 102). With respect to gut microbial structure, Sijunzi Decoction increases beneficial bacteria such as *Bifidobacterium* and *Lactobacillus*, thereby improving the intestinal physical barrier and enhancing microbial metabolite production (**Table 1**). It also increases overall microbial richness. Wang Zhuo et al. (103) established a rat model of gut microbiota dysbiosis using senna leaf and rhubarb. Treatment with Sijunzi Decoction significantly increased gut microbiota diversity in both groups. In addition, Sijunzi Decoction increased SCFAs levels and enhanced local intestinal immunity (104). Studies have also shown that Sijunzi Decoction improves intestinal barrier function through multiple pathways, reduces inflammatory responses, improves insulin resistance, lowers circulating LPS levels, and decreases the release of pro-inflammatory factors, thereby alleviating intestinal barrier damage.

## 6 Limitations and future perspectives

Although growing evidence suggests that natural products exert anti-obesity effects through modulation of the gut microbiota, several challenges remain to be addressed. First, substantial interindividual variability in gut microbiota composition limits the stability and reproducibility of therapeutic responses to natural product interventions. Gut microbial composition is influenced by multiple factors, including genetic background, dietary habits, and lifestyle (108). Therefore, the same natural product may elicit distinct microbial responses among individuals, ultimately resulting in considerable variability in treatment outcomes. Second, most current evidence is derived primarily from animal studies, whereas high-quality clinical evidence remains relatively limited. Third, natural products, particularly TCM formulations, consist of multiple bioactive constituents, and variations in the source of medicinal materials, processing procedures, and dosage regimens may affect their therapeutic efficacy. (109). Fourth, the causal relationship between gut microbiota alterations and anti-obesity effects has not yet been fully elucidated (110). Most existing studies are based on associations between alterations in microbial abundance and improvements in metabolic parameters, whereas direct evidence establishing causality remains limited. Another limitation is that the available evidence is uneven across different categories of natural medicines. Purified natural compounds are generally supported by clearer pharmacological evidence and better-defined mechanisms, whereas herbal extracts, single medicinal herbs, and especially multi-herb TCM formulas exhibit greater compositional complexity, making mechanistic studies and quality standardization more challenging.

Personalized microbiota-based interventions guided by precision medicine may represent an important direction for future research (111). Given the substantial interindividual variation in gut microbial composition, individualized intervention strategies based on baseline microbial characteristics and metabolic phenotypes may improve the therapeutic efficacy of natural products and reduce variability in treatment responses. In addition, organoid technology may provide a valuable platform for investigating the mechanisms underlying the effects of natural products. Organoids, as three-dimensional structures derived from stem cells, can recapitulate key aspects of intestinal architecture and function *in vitro*. In the future, the establishment of gut organoid–microbiota co-culture systems may enable dynamic evaluation of the effects of natural products and their metabolites on intestinal barrier function, immune responses, and microbial interactions, thereby providing a more precise understanding of their mechanisms of action (112). Furthermore, integrating patient-derived organoids (PDOs) with gut microbiota profiling may facilitate the personalized selection of therapeutic strategies and further enhance treatment precision. Finally, large-scale, multicenter randomized controlled trials with long-term follow-up are warranted to further establish the efficacy and safety of gut microbiota-targeted natural products for obesity management.

## 7 Summary

The development and progression of obesity are not merely the result of an imbalance between energy intake and expenditure, but rather reflect complex, multisystem and multilayered dysregulation. As a critical link between external nutrients and host metabolism, the gut microbiota plays a central role in obesity. Individuals with obesity commonly exhibit reduced gut microbial diversity, decreased abundances of beneficial bacteria such as *Lactobacillus* and *Bifidobacterium*, and increased opportunistic pathogens. These alterations further contribute to chronic low-grade inflammation, insulin resistance, and adipose tissue accumulation. Gut microbiota dysbiosis not only affects energy intake and metabolism but also accelerates the progression of overweight and obesity by impairing intestinal barrier function and activating inflammatory pathways. In this context, natural medicines, owing to their multi-target and holistic regulatory properties, have emerged as promising strategies for intervening in the “gut microbiota–metabolism axis.” Components such as polysaccharides, saponins, flavonoids, and alcohol derivatives can serve as prebiotic substrates, providing energy sources for beneficial bacteria (e.g., *Lactobacillus, Bifidobacterium*, and *Faecalibacterium*) and promoting their growth, while inhibiting excessive expansion of opportunistic pathogens (e.g., *Escherichia coli* and *Enterobacteriaceae*), thereby restoring microbial homeostasis. In addition, gut microbiota-derived metabolites act as key mediators underlying the effects of natural medicines. Active components can promote the production of SCFAs such as butyrate and propionate, activate GPR41/GPR43 signaling, and regulate lipid metabolism and energy homeostasis. At the same time, microbial metabolism can alter the bioavailability and pharmacological properties of natural medicine components. Collectively, natural medicines demonstrate unique advantages in the prevention and treatment of obesity and related metabolic diseases by reshaping gut microbiota structure, restoring microecological balance, and regulating inflammatory and energy metabolism signaling pathways.

Although growing evidence suggests that natural products exert anti-obesity effects through modulation of the gut microbiota, several challenges remain. These include substantial interindividual variability in gut microbiota composition, the limited availability of high-quality large-scale clinical studies, and the complexity of natural products, particularly traditional Chinese medicine formulations, in which variations in the source of medicinal materials, processing procedures, and dosage regimens may influence therapeutic outcomes. In addition, direct evidence establishing causal relationships between gut microbiota alterations and anti-obesity effects remains limited. Future studies should move beyond merely describing changes in microbial composition and focus on elucidating the causal mechanisms underlying the “gut microbiota–metabolite–host” interaction network. Meanwhile, emerging approaches, including personalized microbiota-based interventions guided by precision medicine, next-generation microbiome-based therapeutic strategies, and gut organoid–microbiota co-culture systems, may provide new perspectives and translational opportunities for the application of natural products in obesity management through modulation of the gut microbiota.
